# Pathotype characterization of *Aphanomyces euteiches* isolates collected from pea breeding nurseries

**DOI:** 10.3389/fpls.2024.1332976

**Published:** 2024-03-28

**Authors:** Anne Moussart, Clément Lavaud, Caroline Onfroy, Théo Leprévost, Marie-Laure Pilet-Nayel, Christophe Le May

**Affiliations:** ^1^Terres Inovia, Le Rheu, France; ^2^IGEPP, INRAE, Institut Agro, Univ Rennes, Le Rheu, France; ^3^IGEPP, INRAE, Institut Agro, Univ Rennes, Rennes, France

**Keywords:** Aphanomyces root rot, virulence, aggressiveness, differential genotypes, *Pisum sativum*, *Medicago truncatula*

## Abstract

**Introduction:**

*Aphanomyces euteiches* Drechsler is an oomycete pathogen that affects legume crops, causing root rot, a severe disease of peas (*Pisum sativum* L.) worldwide. While significant research progress has been made in breeding pea-resistant varieties, there is still a need for a deeper understanding of the diversity of pathogen populations present in breeding nurseries located in various legume-growing regions around the world.

**Methods:**

We analysed the diversity of 51 pea-infecting isolates of *A. euteiches*, which were recovered from four American (Athena, OR; Le Sueur, MN; Mount Vernon, WA; Pullman, WA) and three French (Riec-sur-Belon, Templeux-le-Guérard, Dijon) resistance screening nurseries. Our study focused on evaluating their aggressiveness on two sets of differential hosts, comprising six pea lines and five *Medicago truncatula* accessions.

**Results:**

The isolates clustered into three groups based on their aggressiveness on the whole pea set, confirming the presence of pathotypes I and III. Pathotype I was exclusive to French isolates and American isolates from Athena and Pullman, while all isolates from Le Sueur belonged to pathotype III. Isolates from both pathotypes were found in Mount Vernon. The *M. truncatula* set clustered the isolates into three groups based on their aggressiveness on different genotypes within the set, revealing the presence of five pathotypes. All the isolates from the French nurseries shared the same Fr pathotype, showing higher aggressiveness on one particular genotype. In contrast, nearly all-American isolates were assigned to four other pathotypes (Us1, Us2, Us3, Us4), differing in their higher aggressiveness on two to five genotypes. Most of American isolates exhibited higher aggressiveness than French isolates within the *M. truncatula* set, but showed lower aggressiveness than French isolates within the *P. sativum* set.

**Discussion:**

These results provide valuable insights into *A. euteiches* pathotypes, against which the QTL and sources of resistance identified in these nurseries displayed effectiveness. They also suggest a greater adaptation of American isolates to alfalfa, a more widely cultivated host in the United States.

## Introduction

*Aphanomyces euteiches* Drechsler is an oomycete pathogen affecting various legume species and causing the devastating root rot of peas (*Pisum sativum* L.) worldwide (Harveson et al., 2021). In Europe, *A. euteiches* was first observed in Norway in 1925 (Sundheim, 1972), and was reported a few years later in France (Labrousse, 1933), where it has been considered as the most important pathogen of peas since 1993 (Didelot and Chaillet, 1995). The pathogen causes root rots, which develop depending on high soil moisture and are optimal between 16°C and 28°C (Papavizas and Ayers, 1974). In favourable conditions, Cunningham and Hagedorn (1962) observed the rapid invasion of the root cortex, as well as the appearance of the sexual stage (oospores), a few days after the infection. The disease often appears early in the spring, affecting young pea plants, and yield losses may be considerable. Oospores were reported to resist adverse conditions, such as alternate freezing and thawing, dry conditions (Sherwood and Hagedorn, 1962), and to survive in soils for 10 to 20 years in the absence of susceptible crops (Pfender and Hagedorn, 1983). *A. euteiches* displays a broad host range within the legume family (Levenfors et al., 2003; Moussart et al., 2008). Initially regarded as exclusively infecting peas (Scott, 1961), *A. euteiches* was later reported as a pathogen that can also attack other legume species, including common bean, broad bean, faba bean, clover, and alfalfa (Pfender and Hagedorn, 1982; Greenhalgh and Merriman, 1985; Lamari and Bernier, 1985; Burnett et al., 1994; Tivoli et al., 2006; Moussart et al., 2008).

Understanding the diversity of pathogenicity within pathogen populations is crucial for optimizing effective strategies for plant disease management. The diversity of pathogenicity within *A. euteiches* populations infecting peas has been documented in grower fields across multiple countries. However, there have been only a few studies that have compared *A. euteiches* populations between countries. No study has yet provided a description of *A. euteiches* populations from contaminated nurseries used for pea resistance screenings. However, such knowledge is essential for understanding the pathogen populations that interact with the resistance sources, loci or breeding lines during the creation and deployment of pea resistant varieties. In addition, there is a lack of knowledge about the diversity of *A. euteiches* populations that infect peas and their adaptation to other commonly grown legume hosts, such as alfalfa (*Medicago sativa*). Better understanding whether populations are adapted to multiple legume hosts could potentially facilitate the transfer of genetic knowledge regarding resistance from one host to another.

In pea, Wicker and Rouxel (2001) initially identified two main pathotypes within a collection of 109 pea-infecting isolates, based on their differential reactions on a set of six pea genotypes (Wicker et al., 2003). Among these isolates, 88 isolates were from France, and 21 originated from Denmark, Sweden, Norway, the USA, Canada and New Zealand. All French isolates were classified as pathotype I and showed a wide range of aggressiveness. In contrast, a distinct pathotype, named pathotype III, was identified among American isolates and characterized by reduced aggressiveness towards the pea genotype MN313. Regardless of their pathotype, all American isolates displayed lower aggressiveness towards peas compared to French isolates. In an additional study including 34 *A. euteiches* isolates of pathotype I collected from the main pea-growing regions in France, Quillévéré-Hamard et al. (2018) detected a moderate level of pathogenicity diversity across various legume hosts. However, some isolates from fields with a history of diversified legume cultivation exhibited specific genetic patterns. More recently, Sivachandra et al. (2021) identified primarily pathotype I among 32 Canadian isolates collected from fields in Saskatchewan and Alberta, and only three isolates of pathotype III. In Europe, Kälin et al. (2022) showed varying levels of disease severity on pea genotypes, caused by ten *A. euteiches* isolates collected from pea fields in four countries (Sweden, Finland, Italy, France) and representing three genetic clusters, but no specific pathotype was identified.

In alfalfa, Malvick and Grau (2001) and Fitzpatrick et al. (1998) described two pathotypes (race 1 and race 2), based on the reaction of *A. euteiches* isolates on three differential lines (Saranac, Waph-1 and Waph-5). In a survey of 30 fields across 18 counties in Illinois, Malvick et al. (2009) highlighted the diversity of *A. euteiches* populations in alfalfa fields. These populations frequently consisted of both races 1 and 2. In addition, Holub et al. (1991) showed that 97% of isolates collected from pea fields in Wisconsin (USA) had the capacity to infect alfalfa. In contrast, only a limited number of isolates from alfalfa fields displayed pathogenicity towards peas. Wicker et al. (2001) also demonstrated that among 91 pea-infecting isolates from France, the majority were pathogenic on alfalfa. The wide host range of pea-infecting isolates of *A. euteiches* has led several authors to hypothesize that crop rotations involving peas and other legumes such as alfalfa, could potentially facilitate the emergence of complex pathogen populations consisting of multiple pathotypes, promoting population adaptation to different legume hosts (Malvick et al., 1998; Wicker and Rouxel, 2001; Wicker et al., 2001; Levenfors and Fatehi, 2004). The model legume *M. truncatula* is also susceptible to *A. euteiches* infection. Moussart et al. (2007) identified a continuum of variation, ranging from resistance to susceptibility, among different accessions of *M. truncatula* when exposed to a single pea isolate of *A. euteiches*. A similar variation was also observed in *M. truncatula* accessions evaluated for resistance to alfalfa race 2 isolates of *A. euteiches* (Vandemark and Grunwald, 2004). Resistance was shown to be either controlled by a major locus (Pilet-Nayel et al., 2009; Bonhomme et al., 2014), or by a complex genetic network of minor QTL (Hamon et al., 2010), depending on the *A. euteiches* pea or alfalfa pathotype considered. These observations led to hypothesize that the model species *M. truncatula* may constitute an efficient bridge for comparing the expression and genetic control of resistance to *A. euteiches* between grain and forage legumes (Tivoli et al., 2006).

Thus, various studies have characterized pathotypes of pea- or alfalfa- infecting isolates of *A. euteiches* isolated from commercial pea fields. However, the characterization of *A. euteiches* isolates found in breeding nurseries has remained unreported, despite its significance in the development of resistant varieties effective against *A. euteiches* populations within commercial legume fields. To address this gap, a transatlantic collection of 51 A*. euteiches* isolates collected from French and American breeding nurseries was established. These nurseries were grown with pea research genetic material employed to detect Quantitative Trait Loci (QTL) for resistance to *A. euteiches* (Hamon et al., 2013; Desgroux et al., 2016). Genetic structure analysis of this collection using Sequence-Related Amplified Polymorphism (SRAP) (Le May et al., 2018) or Simple Sequence Repeat (SSR) (Mieuzet et al., 2016) markers clustered the French and American isolates in two different groups, showing higher genetic diversity between countries than within them. However, this collection has not yet been characterized for its pathogenicity diversity.

The objective of this study was to characterize the pathotypes of *A. euteiches* isolates from the transatlantic collection, focusing on their aggressiveness and virulence towards both pea and *M. truncatula*. Two questions were addressed: (i) are the pathotypes found in French and American nurseries consistent with those employed in genetics and breeding programs? (ii) are American isolates better adapted to *Medicago* spp., a legume species more extensively cultivated in the USA than in France? In this study, we evaluated the aggressiveness and virulence of the 51 A*. euteiches* isolates from the transatlantic collection established by Le May et al. (2018) on a set of pea differential genotypes, as defined by Wicker et al. (2001), and on a new set of *M. truncatula* accessions specifically curated for this work.

## Materials and methods

### Pathogen material

The 51 isolates of *A. euteiches* employed in this study were collected from contaminated nurseries in 2005 and subsequently baited following the protocol presented in Le May et al. (2018). The set included 25 isolates originating from three French nurseries, *i.e.* ten isolates from Dijon (isolates Di1 to Di10), ten isolates from Templeux-le-Guérard (Tpx1 to Tpx10) and five isolates from Riec-sur-Belon (Ri2, Ri4, Ri7, Ri8 and Ri10). In addition, this study included 26 isolates from four nurseries in the United States (US)*, i.e.* seven isolates from Athena (Ath1 to Ath7), nine isolates from Le Sueur (LS1 to LS3, LS5 to LS10), five isolates from Mount-Vernon (MV1, MV3 to MV5 and MV7) and five isolates from Pullman (Plm1 to Plm4 and Plm7). The nurseries were characterized based on distinct growing seasons and climatic conditions (**Additional File 1**). All 51 isolates were single-spored, grown, and maintained on Corn Meal Agar (CMA) at a temperature of 10°C. Two isolates were employed as standards in this study: RB84, originating from a pea field in Riec-sur-Belon, France, selected as the reference isolate for pathotype I (Moussart et al., 2007); and Ae109, also known as synonym 467, collected in Wisconsin, USA, used as the reference isolate for pathotype III (Malvick et al., 1998; Wicker and Rouxel, 2001).

### Plant material

The pea differential set previously established by Wicker et al. (2003), was used in this study to distinguish the two main pathotypes I and III, according to the differential reaction of the MN313 genotype. The pea set consisted of a total of six genotypes, including (i) the spring-sown field pea cultivars Baccara (Ets Florimond Desprez) and Capella (Svalöf Weïbull AB), (ii) the garden pea breeding lines MN313 (Davis et al., 1995), 552 (Gritton, 1995) and 90-2131 (Kraft, 1992), and (iii) the germplasm accession PI180693 (USDA Plant Introduction Pullman).

The *M. truncatula* differential set consisted of a total of five genotypes, including A17 (Australia), DZA045.5 (Algeria), F83005.5 (France), DZA241.2.2 (Algeria) and F83005.9 (France) (Moussart et al., 2007). The establishment of the *M. truncatula* differential set involved a two-step process. Initially, a screening of 112 *M. truncatula* pure lines (obtained from Dr Prosperi, INRAE Montpellier, UMR AGAP, Mauguio, France) using the reference isolate RB84 (Pilet-Nayel et al., 2005) led to the selection of a subset of 15 accessions based on their differential responses. Then, these 15 *M. truncatula* accessions were screened using both reference isolates RB84 and Ae109, according to the procedure presented in Moussart et al. (2007), resulting in the selection of five genotypes. Notably, Ae109 was more aggressive on all the five genotypes than RB84. DZA045.5 and F83005.5 were partially resistant and susceptible to both isolates, respectively. DZA241.2, A17 and F83005.9 showed differential reactions to both isolates (unpublished results).

### Pathogenicity tests

Pathogenicity experiments were conducted at INRAE, IGEPP (Le Rheu, France). In these experiments, all isolates were tested on both the pea and *M. truncatula* differential sets within the same growth chamber.

For the pea differential sets comprising both pea and *M. truncatula* genotypes, the methods described by Moussart et al. (2001) and Moussart et al. (2007), were followed, respectively. Zoospores were produced following the method described by Wicker et al. (2001) for the French isolates. However, for the American isolates that did not yield sufficient zoospores under these conditions, we made adjustment to the culture process. Specifically, we transferred six agar discs from four-day-old cultures on CMA to glucose-peptone broth. These discs were then incubated at 22°C (instead of 25°C) for a period of three days before being rinsed with sterilized Volvic^®^ water, following the same rinsing procedure as described above.

Pea and *M. truncatula* seeds were planted in 500 ml plastic pots (5 plants/pot) filled with vermiculite (VERMEX, M, Soprema, France). Each pot was considered as an experimental unit and there were three replicates per isolate x host combination in each independent experiment. Two independent experiments were performed for each isolate. A total of 30 plants (5 plants * 3 replicates * 2 independent experiments) was evaluated per isolate x host combination. Pots were arranged in a completely randomised design within a growth-chamber under controlled conditions, maintaining temperatures ranging within 23-25°C and a photoperiod of 14 h. Seven days after sowing, seedlings were inoculated by applying 5 ml of inoculum suspension at the base of each plant (10^3^ zoospores per plant on pea; 10^4^ zoospores per plant on *M. truncatula*) using a pipette. After inoculation, the vermiculite was saturated with water to favour disease development. Pea and *M. truncatula* plants were removed 7 and 14 days after inoculation, respectively, and disease severity was visually evaluated on infected roots using a scoring scale ranging from 0 to 5 scoring, as previously described by Moussart et al. (2007): 0 = no symptoms; 1 = traces of discoloration on the roots (<25%); 2 = discoloration of 25 to 50% of the roots; 3 = discoloration of 50 to 75% of the roots; 4 = discoloration of more than 75% of the roots; 5 = dead plant.

### Data analysis

Statistical analyses of variance were conducted using R software (R version 4.3.1; R Core Team, 2023). Disease severity data obtained from the pea and *M. truncatula* differential sets were analysed separately, using a linear mixed model [LMM; ‘lmer’ function (Bates et al., 2015)], considering the disease severity as the explanatory variable, the genotype, the isolate, and the genotype x isolate interaction as fixed factors, and the experiment effect as random factor. Estimated Marginal Mean (EMMean) values were estimated for each genotype and isolate combination using the ‘emmeans’ function (Lenth, 2023). Multiple comparisons of EMMean values were performed (i) between pea or *M. truncatula* genotypes for each isolate, and (ii) between isolates for each nursery based on the mean response of each set of pea or *M. truncatula* genotypes, with the Tukey test (α=5%), using the ‘cld’ function (Graves et al., 2019). For each isolate, phenotypes of pathogenicity were defined based on significant differences of disease severity values between the six pea genotypes or between the five *M. truncatula* lines. The effect of the country of origin of the isolates on the mean disease severity observed on each set of pea or *M. truncatula* genotypes was tested using a general linear model [LM; ‘lm’ function (Chambers, 1992)], with the disease severity as the explanatory variable and the country as fixed factor.

Principal component analysis (PCA) and hierarchical clustering analysis (HCA) were performed based on EMMean values obtained separately from pea and *M. truncatula* genotypes, using R software. PCA was conducted to analyse similarities of i=53 or 52 isolates of *A. euteiches* (the analysis included Ae109 and RB84 reference isolates; the LS9 isolate was not tested with the *M. truncatula* differential set of genotypes), for pea or *M. truncatula* data, respectively. The ‘PCA’ function implemented in the ‘FactoMineR’ package was used for this analysis (Lê et al., 2008). HCA was performed to define different clusters of isolates using the Ward D method aiming to minimize the variance within each defined cluster (Murtagh and Legendre, 2014). HCA was implemented using the ‘dist’ and ‘hclust’ functions from R.

## Results

### Aggressiveness and virulence of the *A. euteiches* isolates on the pea differential set

The pea differential set allowed the identification of the two distinct pathotypes I and III within the collection of 51 isolates of *A. euteiches*. This identification was based on the varying responses observed in the pea genotype MN313. Isolates belonging to the pathotype I displayed aggressiveness across the entire set of genotypes. In contrast, isolates belonging to the pathotype III showed lower aggressiveness when interacting with the genotype MN313 (**Additional File 2A**). Disease severity on MN313 was significantly lower (*P* < 0.001) compared to that on Baccara and Capella, the most susceptible genotypes. In addition, it was either equal to or lower than the disease severity observed on PI180693, the most resistant genotype among the set.

Among the 25 isolates obtained from French nurseries, 22 isolates belonged to the pathotype I (**Table 1**). Three isolates (Di6, Di7 and Di9) were not assigned to a pathotype group since they displayed intermediate behaviours that fell between the characteristics of pathotypes I and III. For two of the three isolates, disease severity values on MN313 (2.8 and 2.1 for Di6 and Di7, respectively) were higher than what is typically observed for isolates belonging to pathotype III. For Di9 isolate, disease severity on Capella (1.8) was lower than generally observed for isolates belonging to pathotype III. In addition, these values were significantly lower than those recorded on Baccara and Capella, and matched the disease severity observed on PI180693, as observed for isolates belonging to pathotype I. Significant variability in the mean disease severity across the entire set of pea genotypes was observed among the French isolates (*P* < 0.001). For each isolate, the range of disease severity observed across the six pea genotypes was low (0.6 < Range_DS_ < 1.2) but significant (*P* < 0.001), except for five isolates from Templeux-le-Guérard (Tpx1, Tpx6, Tpx7, Tpx8 and Tpx10; 1.5 < Range_DS_ < 2.2) and one isolate from Dijon (Di9; Range_DS_ = 1.4), exhibiting larger range of disease severity but lower mean level of aggressiveness.

**Table 1:** Disease severity on six pea differential host genotypes, for 51 *A. euteiches* isolates and two *A. euteiches* reference isolates (RB84 and Ae109), from French and American nurseries.

Nursery	Isolate	Genotype ^a^						Pathotype ^b^	EMMeans Disease Severity ^c^	HAC groups ^d^
		Baccara	Capella	MN313	552	90-2131	PI180693			
Dijon (FR)	Di1	3.4 a	3.3 ab	3.0 bc	3.1 ab	2.5 d	2.7 cd	I	3.0 CDE	2
	Di2	3.7 a	3.3 b	3.0 bc	2.7 c	2.8 c	2.7 c	I	3.0 CDE	2
	Di3	3.8 a	3.3 b	3.1 bc	2.9 c	2.9 c	2.9 c	I	3.1 E	2
	Di4	3.5 a	3.0 b	2.8 bc	2.6 c	2.7 bc	2.6 c	I	2.9 C	2
	Di5	3.4 a	3.4 a	3.3 a	2.7 b	2.8 b	2.9 b	I	3.1 DE	2
	Di6	3.5 a	3.3 a	2.8 b	2.5 c	2.8 bc	2.8 bc	NA	3.0 CD	2
	Di7	3.0 a	2.5 b	2.1 cd	2.2 bc	1.8 d	2.1 cd	NA	2.3 B	1
	Di8	3.6 a	3.1 b	3.0 bc	2.8 c	2.8 bc	2.7 c	I	3.0 CDE	2
	Di9	2.8 a	1.8 b	1.4 c	1.5 bc	1.7 bc	1.6 bc	NA	1.8 A	1
	Di10	3.5 a	3.1 b	3.1 b	2.9 b	2.9 b	2.9 b	I	3.1 DE	2
Riec-sur-Belon (FR)	Ri2	3.8 a	3.2 bc	3.3 b	3.2 bc	2.9 c	2.9 c	I	3.2 BC	2
	Ri4	3.8 a	3.3 b	3.7 a	3.0 b	3.0 b	3.0 b	I	3.3 C	2
	Ri7	3.8 a	3.4 b	3.5 ab	3.1 bc	2.9 c	2.8 c	I	3.3 C	2
	Ri8	3.6 a	3.3 ab	3.3 ab	3.1 b	2.9 b	2.5 c	I	3.1 AB	2
	Ri10	3.5 a	3.2 a	3.3 a	2.7 b	2.5 b	2.8 b	I	3.0 A	2
Templeux- Le-Guérard (FR)	Tpx1	3.6 a	3.2 b	2.7 c	1.9 d	1.6 d	1.8 d	I	2.5 A	2
	Tpx2	3.3 a	3.0 ab	2.9 b	2.7 b	2.2 c	2.1 c	I	2.7 BC	2
	Tpx3	3.6 a	3.2 ab	3.2 bc	2.9 c	2.9 bc	2.9 c	I	3.1 EF	2
	Tpx4	3.5 a	3.0 b	2.7 bc	2.4 cd	2.4 d	2.5 cd	I	2.8 CD	2
	Tpx5	3.7 a	3.2 b	3.3 b	3.0 b	2.7 c	2.7 c	I	3.1 E	2
	Tpx6	3.3 a	2.8 b	3.2 a	2.5 b	1.6 c	1.7 c	I	2.5 AB	2
	Tpx7	3.6 a	3.4 a	3.4 a	2.8 b	2.0 c	2.1 c	I	2.9 D	2
	Tpx8	3.7 a	3.8 a	3.5 a	2.4 b	2.3 b	1.6 c	I	2.9 D	2
	Tpx9	3.7 a	3.6 a	3.5 a	3.1 b	3.1 b	2.7 c	I	3.3 F	2
	Tpx10	3.8 a	3.3 b	3.3 b	2.5 c	2.2 cd	1.8 d	I	2.8 CD	2
Athena (US)	Ath1	3.3 a	2.9 bc	3.1 ab	2.9 bc	2.7 cd	2.3 d	I	2.9 E	2
	Ath2	2.9 a	3.0 a	2.8 a	2.8 a	1.8 b	1.4 b	I	2.4 C	2
	Ath3	3.8 a	3.3 b	3.2 bc	3.1 bc	2.9 c	2.8 c	I	3.2 F	2
	Ath4	3.0 a	3.0 a	2.7 ab	2.5 bc	2.3 c	2.3 c	I	2.7 D	2
	Ath5	3.1 a	3.0 ab	2.8 b	2.2 c	1.9 d	1.7 d	I	2.4 C	2
	Ath6	3.1 a	2.9 a	2.1 b	1.9 bc	1.6 cd	1.4 d	I	2.2 B	1
	Ath7	2.8 a	2.1 b	1.8 bc	1.6 cd	1.3 de	1.0 e	I	1.8 A	1
Le Sueur (US)	LS1	2.7 a	2.2 b	0.9 d	1.9 b	1.4 c	1.4 c	III	1.8 C	1
	LS2	3.2 a	3.0 a	1.5 c	2.2 b	1.6 c	1.5 c	III	2.1 DE	1
	LS3	3.1 a	2.8 a	1.1 d	2.2 b	1.8 c	1.5 c	III	2.1 D	1
	LS5	3.3 a	3.0 a	1.7 cd	2.5 b	1.9 c	1.4 d	III	2.3 EF	1
	LS6	1.6 a	1.5 ab	1.0 c	1.0 c	1.2 bc	1.0 c	NA	1.2 A	1
	LS7	3.4 a	2.9 b	1.5 d	2.3 c	1.6 d	1.3 d	III	2.1 DE	1
	LS8	3.1 a	2.8 a	1.3 c	1.9 b	1.9 b	1.5 c	III	2.1 D	1
	LS9	2.4 a	2.4 a	0.4 d	1.8 b	1.0 c	1.3 bc	III	1.6 B	1
	LS10	3.1 a	3.0 ab	1.5 d	2.6 b	2.3 c	2.2 c	III	2.5 F	1
Mount Vernon (US)	MV1	2.9 a	2.0 b	1.8 bc	1.5 c	1.5 c	1.5 c	I	1.8 A	1
	MV3	3.2 a	2.8 a	0.9 c	2.2 b	2.0 b	2.2 b	III	2.2 B	1
	MV4	2.9 a	3.0 a	1.1 c	2.3 b	2.3 b	2.0 b	III	2.3 B	1
	MV5	3.0 a	2.8 a	2.3 b	1.9 c	1.8 c	2.0 c	I	2.3 B	1
	MV7	3.7 a	3.3 b	3.0 bc	2.7 cd	2.6 d	2.9 cd	I	3.0 C	2
Pullman (US)	Plm1	3.0 a	2.7 a	2.2 b	2.0 b	1.1 c	1.1 c	I	2.0 A	1
	Plm2	3.4 a	3.2 a	3.1 a	2.3 b	1.5 c	1.3 c	I	2.5 B	2
	Plm3	3.3 a	3.1 a	2.7 b	2.7 b	2.3 c	1.6 d	I	2.6 C	2
	Plm4	3.4 a	3.1 ab	2.8 bc	2.6 c	1.6 d	1.5 d	I	2.5 BC	2
	Plm7	3.2 a	3.1 a	2.9 a	3.0 a	2.4 b	2.2 b	I	2.8 D	2
Standard isolates	RB84	3.7 a	3.4 b	3.3 b	3.0 c	2.7 d	2.4 e	I	3.1	2
	Ae109	3.3 a	2.9 b	1.2 d	2.1 c	2.1 c	1.8 c	III	2.2	1

Disease severity was recorded on a scale from 0 (no symptoms) to 5 (dead plant). ^a^ For each isolate (*i.e.* each row), EMMean values on the different pea genotypes followed by the same lower case letter are not significantly different (Tukey test, α=5%). ^b^ Pathotypes identified according to Wicker et al. (2003) and Wicker and Rouxel (2001); NA: isolates with undefined pathotypes. ^c^ Between isolates for each nursery, EMMean values on all pea genotypes followed by the same upper-case letter are not significantly different (Tukey test, α=5%). ^d^ Hierarchical Ascending Classification groups obtained from the Ward D method in this study.

**Figure 1:**
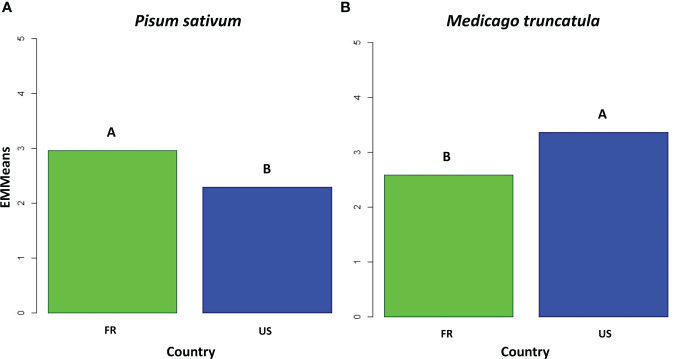
Bar plot of disease severity adjusted means for the French (FR) and American (US) isolates of the transatlantic collection of *A. euteiches* and two *A. euteiches* reference isolates (n=52), **(A)** on the six pea differential genotypes, and **(B)** the five *Medicago truncatula* differential genotypes. Significantly different means are indicated by letters (Tukey test, α = 1%).

Among the 26 isolates obtained from American nurseries, 15 were classified as belonging to pathotype I (**Table 1**). One isolate (LS6) was not assigned to a pathotype group since it displayed intermediate behaviour between the characteristics of pathotype I and III. For LS6 isolate, the disease severity on Capella (1.5) was lower than that usually observed for pathotype III isolates. The remaining ten isolates belonged to pathotype III, showing significantly lower disease severity on MN313 than those observed on Baccara and Capella. Additionally, their disease severity matched or was even lower than that on PI180693, as displayed by the reference isolate Ae109. All the isolates from Athena and Pullman nurseries belonged to pathotype I. All the isolates from Le Sueur (except LS6) belonged to pathotype III. However, in the case of Mount-Vernon isolates, there was a split: some were categorized as pathotype I (MV1, MV5, and MV7), while others fell into pathotype III (MV3 and MV4). The majority of American isolates distinguished between susceptible and partially resistant genotypes within the differential set. Significant variability for mean disease severity across the entire set of pea genotypes was observed within isolates from the United States. Four American isolates, including Ath7 and MV1 from pathotype I, as well as LS1 and LS9 from pathotype III, showed low mean disease severity values (≤1.8) over the whole set of pea genotypes. Conversely, isolates Ath3 and MV7 showed high mean disease severity values (≥3) across the entire set. Overall, the American isolates showed a lower level of aggressiveness compared to the French isolates when tested on the set of pea genotypes (**Figure 1**).

**Figure 2:**
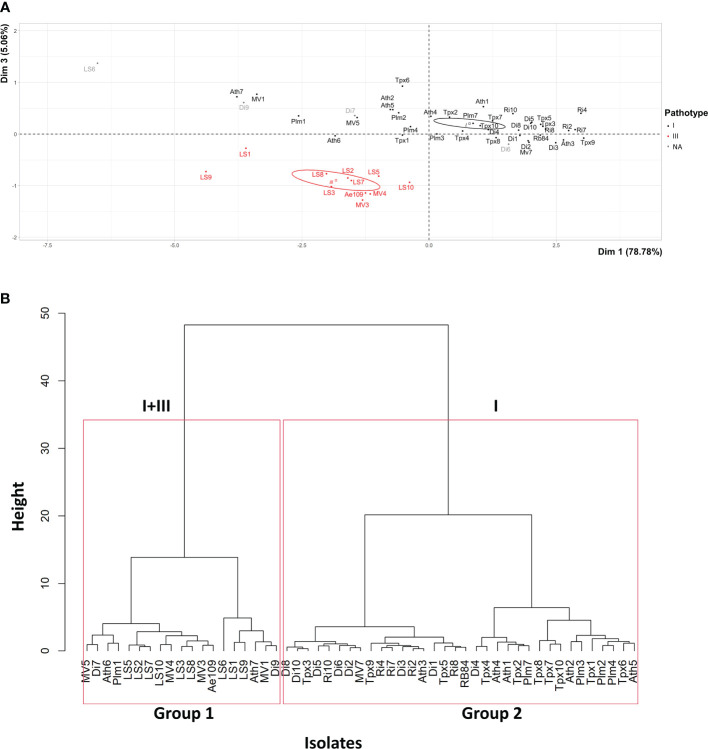
**(A)** Principal Component Analysis and **(B)** Hierarchical Ascending Classification, of 51 *A. euteiches* isolates from the US-FR transatlantic collection and two *A. euteiches* reference isolates (RB84 and Ae109), based on disease severity data on six pea differential genotypes. I, III: pathotypes I and III of *A. euteiches*. NA, isolates with undefined pathotype. Confidence ellipses, calculated at the confidence level of 95%, are overlaid to visualize the grouping patterns.

PCA mainly distinguished the 51 isolates based on their level of aggressiveness across the entire set of pea genotypes, as shown by the high percentage of total variation explained by the first principal component (PCA.Dim1: 78.78%) (**Figure 2A**). Isolates classified as pathotype I exhibited a higher level of aggressiveness when evaluated on the differential set of pea genotypes in comparison to isolates belonging to pathotype III. The second (PCA.Dim2: 9.76%) and third (PCA.Dim3: 5.06%) principal components of the analysis separated isolates based on their aggressiveness towards susceptible versus partially resistant pea genotypes and the MN313 genotype, respectively. Variability in aggressiveness was observed among pathotypes I and III for susceptible and partially resistant genotypes (Dim2, not shown). Isolates from pathotype III clustered based on aggressiveness towards MN313 (Dim3, **Figure 2A**).

HAC identified two groups among the 51 isolates, according to their mean level of aggressiveness across the set of pea genotypes, which aligns with the results from the PCA (**Figure 2B**). Group 1 comprised 18 isolates, along with the standard Ae109 isolate. This group encompassed 16 isolates from American nurseries and two isolates from French nurseries. This group also included six isolates, mostly collected from American nurseries, displaying lower levels of aggressiveness. Among these isolates, two were from pathotype I (Ath7, MV1), two were from pathotype III (LS1, LS9), and two isolates from French nurseries (LS6, Di9) were unassigned to a pathotype. Group 2 included 33 isolates, along with the standard RB84 isolate. All isolates were categorized as belonging to pathotype I. This group encompassed 23 isolates from French nurseries and 10 isolates from American nurseries.

### Aggressiveness and virulence of the *A. euteiches* isolates on the *M. truncatula* differential set

All the 25 isolates from the French nurseries, as well as the reference isolate RB84, belonged to the same pathotype, named Fr, characterized by significantly higher disease severity on F83005.5 compared to the other four genotypes within the *M. truncatula* differential set (**Table 2**; **Additional File 2B**). Significant variability (*P<0.001*) was observed among the isolates in terms of mean disease severity across the five genotypes. For each isolate, the range of disease severity across the five *M. truncatula* genotypes varied from low to high (0.1 < Range_DS_ < 2.3), with all French isolates compared to only half of the American isolates showing higher ranges (Range_DS_ ≥ 1.4).

**Table 2:** Disease severity on five *M. truncatula* differential host accessions, for 50 *A. euteiches* isolates and two *A. euteiches* reference isolates (RB84 and Ae109), from French and American nurseries.

Nursery	Isolate	Accession ^a^					Pathotype ^b^	EMMeans disease severity ^c^	HAC group ^d^
		F83005.5	F83005.9	A17	DZA241.2	DZA045.5			
Dijon (FR)	Di1	3.9 a	2.7 b	2.7 b	1.8 c	1.8 c	Fr	2.6 BCDE	3
	Di2	3.6 a	2.4 b	1.9 c	1.9 c	1.9 c	Fr	2.3 A	3
	Di3	3.5 a	2.2 c	2.9 b	2.0 c	2.0 c	Fr	2.5 BCD	3
	Di4	3.8 a	2.8 b	2.0 c	1.9 c	1.9 c	Fr	2.5 ABC	3
	Di5	3.8 a	3.0 b	1.9 c	1.8 c	1.8 c	Fr	2.4 AB	3
	Di6	4.0 a	2.9 b	2.4 c	2.3 c	1.9 d	Fr	2.7 DE	3
	Di7	3.6 a	3.1 b	2.7 c	2.2 d	2.0 d	Fr	2.7 E	3
	Di8	3.4 a	2.5 b	2.6 b	2.6 b	2.0 c	Fr	2.6 BCDE	3
	Di9	3.4 a	3.0 b	2.7 b	2.0 c	2.0 c	Fr	2.6 CDE	3
	Di10	3.7 a	3.0 b	3.0 b	2.2 c	1.9 c	Fr	2.7 E	3
Riec-sur-Belon (FR)	Ri2	4.0 a	3.1 c	3.5 b	2.0 d	1.9 d	Fr	2.9 D	3
	Ri4	3.9 a	2.9 b	2.8 b	1.9 c	1.9 c	Fr	2.7 C	3
	Ri7	3.8 a	2.3 c	2.8 b	1.9 d	1.9 d	Fr	2.6 B	3
	Ri8	3.5 a	2.0 b	2.0 b	1.8 b	2.0 b	Fr	2.3 A	3
	Ri10	4.0 a	2.3 b	2.1 bc	1.9 c	1.9 c	Fr	2.5 B	3
Templeux-Le-Guérard (FR)	Tpx1	4.0 a	2.5 b	2.6 b	1.9 c	1.9 c	Fr	2.6 BC	3
	Tpx2	3.9 a	2.7 b	2.4 c	1.7 e	2.0 d	Fr	2.6 ABC	3
	Tpx3	4.0 a	3.0 b	2.2 c	1.8 d	1.9 cd	Fr	2.6 BC	3
	Tpx4	3.8 a	2.3 b	2.1 bc	1.9 c	2.0 bc	Fr	2.4 A	3
	Tpx5	4.0 a	2.8 b	1.9 c	1.8 c	1.7 c	Fr	2.4 AB	3
	Tpx6	3.5 a	2.9 b	2.2 c	1.8 d	1.7 d	Fr	2.4 AB	3
	Tpx7	3.6 a	3.1 b	2.2 c	1.7 d	1.8 d	Fr	2.5 ABC	3
	Tpx8	3.9 a	3.1 b	2.2 c	1.9 d	2.0 cd	Fr	2.6 C	3
	Tpx9	4.0 a	2.9 b	2.4 c	1.8 d	2.1 c	Fr	2.6 C	3
	Tpx10	4.0 a	3.2 b	2.8 c	1.9 d	2.2 d	Fr	2.8 D	3
Athena (US)	Ath1	3.7 ab	3.6 b	4.0 a	4.0 a	3.1 c	Us2	3.7 D	1
	Ath2	3.2 b	3.3 b	4.0 a	4.0 a	2.4 c	Us3	3.4 C	1
	Ath3	2.9 a	2.6 a	2.3 b	1.9 c	1.9 bc	NA	2.3 A	2
	Ath4	3.9 a	3.5 b	4.0 a	4.0 a	2.8 c	Us2	3.6 D	1
	Ath5	3.7 b	3.1 c	4.0 a	3.9 ab	2.5 d	Us2	3.4 C	1
	Ath6	3.2 b	3.0 b	4.0 a	3.8 a	2.4 c	Us3	3.3 C	1
	Ath7	2.4 b	2.5 b	4.0 a	3.7 a	2.2 b	Us4	3.0 B	2
Le Sueur (US)	LS1	3.9 a	3.5 b	4.0 a	3.8 a	2.4 c	Us2	3.5 B	1
	LS2	4.0 a	3.9 a	4.0 a	3.8 ab	3.6 b	Us1	3.9 DE	1
	LS3	4.0 a	3.9 a	3.9 a	3.9 a	2.8 b	Us2	3.7 C	1
	LS5	4.0 a	3.8 ab	4.0 a	3.9 ab	3.6 b	Us1	3.9 DE	1
	LS6	2.5 b	2.1 c	3.6 a	3.8 a	1.9 c	Us4	2.8 A	2
	LS7	4.2 a	4.0 a	4.0 a	4.0 a	2.7 b	Us2	3.8 CD	1
	LS8	4.0 a	3.9 a	4.0 a	4.0 a	2.7 b	Us2	3.7 C	1
	LS10	4.0 a	4.0 a	4.0 a	4.0 a	3.9 a	Us1	4.0 E	1
Mount Vernon (US)	MV1	2.8 a	2.0 b	2.0 b	1.9 b	2.0 b	Fr	2.1 A	2
	MV3	3.9 a	3.5 b	3.9 a	3.8 ab	3.0c	Us2	3.6 C	1
	MV4	4.0 a	3.8 a	4.0 a	4.0 a	3.1 b	Us2	3.8 C	1
	MV5	2.3 a	2.3 ab	2.0 bc	1.9c	2.2 abc	NA	2.1 A	2
	MV7	3.3 a	2.8 b	2.5 b	2.0 c	2.0 c	Fr	2.5 B	3
Pullman (US)	Plm1	3.5 b	3.1 c	4.0 a	3.9 a	2.2 d	Us3	3.3 A	1
	Plm2	3.1 c	3.4 b	4.0 a	4.0 a	2.3 d	Us3	3.4 A	1
	Plm3	3.6 b	3.5 b	4.0 a	4.0 a	2.9 c	Us3	3.6 B	1
	Plm4	4.0 a	3.9 a	4.0 a	4.0 a	2.7 b	Us2	3.7 C	1
	Plm7	4.0 a	3.4 b	4.0 a	3.9 a	2.2 c	Us2	3.5 B	1
Standard isolates	RB84	4.0 a	2.8 b	2.7 c	2.0 d	1.8 e	Fr	2.7	3
	Ae109	4.1 a	3.9 ab	3.7 b	3.8 b	2.8 c	Us2	3.6	1

Disease severity was recorded on a scale from 0 (no symptoms) to 5 (dead plant). ^a^ For each isolate (*i.e.* each row), EMMean values on the different *M. truncatula* genotypes followed by the same lower case letter are not significantly different (Tukey test, α=5%). ^b^ Pathotypes identified according to this study; NA: isolates with undefined pathotypes. ^c^ Between isolates for each nursery, EMMean values on all *M. truncatula* genotypes followed by the same upper-case letter are not significantly different (Tukey test, α=5%). ^d^ Hierarchical Ascending Classification groups obtained from the Ward D method in this study.

The *M. truncatula* differential set was not challenged by the American isolate LS9 due to the inability to produce a sufficient quantity of zoospores from this isolate for inoculation at the required concentration. The 25 isolates from the American nurseries were classified into five distinct pathotypes, named Fr, Us1, Us2, Us3, and Us4, according to their aggressiveness towards the *M. truncatula* differential set (**Table 2**; **Additional File 2B**). (i) Isolates from pathotype Us1 (3 isolates: LS2, LS5 and LS10) exhibited high aggressiveness across the entire set, with no significant differences observed in disease severity between the most resistant genotype DZA045.5, and at least one of the four other genotypes. (ii) Pathotype Us2 (11 isolates: Ath1, Ath4, Ath5, LS1, LS3, LS7, LS8, MV3, MV4, Plm4, Plm7) was characterized by disease severity on DZA045.5 significantly lower than on the four other genotypes, and at least one genotype between F83005.5 and F83005.9 (intermediate behavior) that was not significantly different to at least one genotype between A17 and DZA241.2 (susceptible). (iii) Pathotype Us3 (5 isolates: Ath2, Ath6, Plm1, Plm2, Plm3) was characterized by disease severity on DZA045.5 significantly lower than on the four other genotypes, and both genotypes F83005.5 and F83005.9 that were significantly different to both genotypes A17 and DZA241.2. (iv) Pathotype Us4 (2 isolates: Ath7 and LS6) was characterized by disease severity on DZA045.5, not significantly lower than at least one genotype between F83005.5 and F83005.9, but significantly lower than both genotypes DZA241.2 and A17 (*p-value* < 0,001). Two isolates, MV1 and MV7, were assigned to pathotype Fr. The last two isolates, Ath3 and MV5, could not be classified, but their behaviour closely resembled the pattern exhibited by isolates from pathotype Fr.

Isolates from pathotypes Us1, Us2 and Us3 were significantly more aggressive (*P* < 0.001) on the *M. truncatula* differential set compared to isolates from pathotypes Fr and Us4. Mean disease severity among American isolates was higher (2.8 < MeanDS < 4) than that observed among French isolates (2.3 < MeanDS < 2.9), except for MV1, MV5, MV7, and Ath3 isolates. The level of partial resistance of DZA045.5 was lower for the American isolates (1.9 < DS < 3.9) than for the French isolates (1.7 < DS < 2.2). Significant variations in mean disease severity were observed among isolates from each of the Athena (US), Le Sueur (US), Mount Vernont (US) and Riec-sur-belon (FR) nurseries. However, such variations were not observed among isolates from Dijon (FR), Templeux-le-Guérard (FR), or Pullman (US) nurseries.

**Figure 3:**
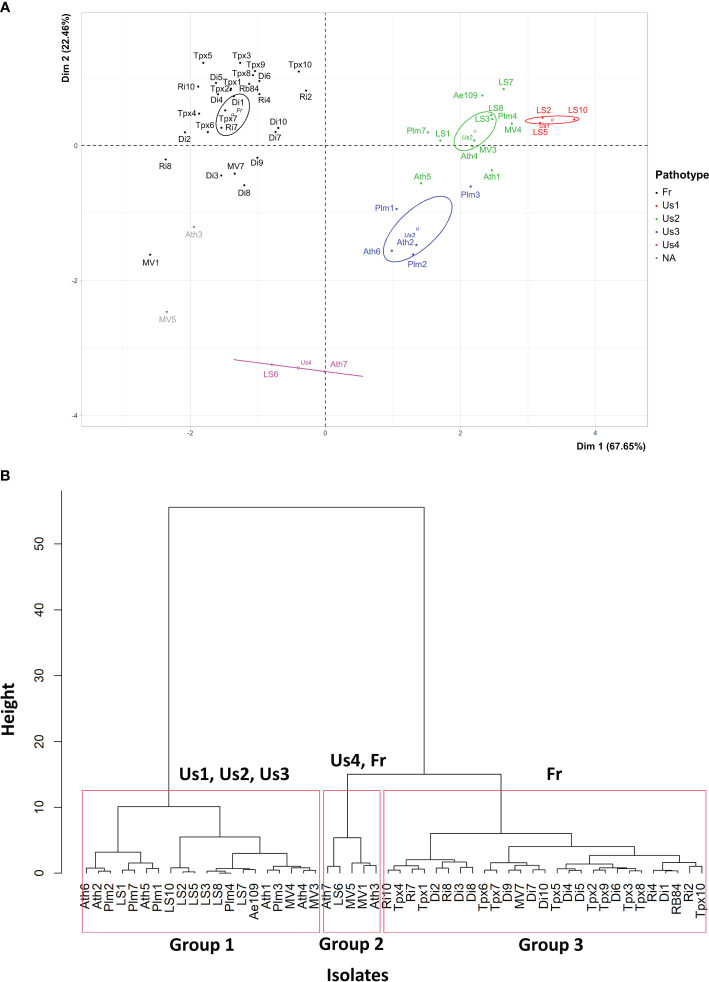
**(A)** Principal Component Analysis and **(B)** Hierarchical Ascending Classification, of 50 *A. euteiches* isolates from the US-FR transatlantic collection and two *A. euteiches* reference isolates (RB84 and Ae109), based on disease severity data on five *Medicago truncatula* differential genotypes. Us1, Us2, Us3, Us4, Fr: pathotypes Us1, Us2, Us3, Us4 and Fr of *A. euteiches*. NA: isolates with undefined pathotype. Confidence ellipses, calculated at the confidence level of 95%, are overlaid to visualize the grouping patterns.

PCA mainly separated the 50 isolates based on their aggressiveness towards the four *M. truncatula* genotypes: DZA045.5, F83005.9, A17 and DZA241.2, as shown by the high percentage of total variation explained by the first principal component (PCA.Dim1: 67.65%) (**Figure 3A**). The isolates belonging to the American pathotypes (except Us4) differed from the French isolates by their high level of aggressiveness on the four genotypes. The second and third principal components of the analysis separated the isolates based on their aggressiveness on the F83005.5 and DZA045.5 genotypes, respectively (PCA.Dim2: 22.46%; PCA.Dim3: 5.59%). The isolates belonging to the Us3 and Us4 pathotypes differ from those belonging to the Fr, Us1 and Us2 pathotypes by their lower aggressiveness on F83005.5 (Dim2, **Figure 3A**). The isolates from the Us1 pathotype differ from the others based on their higher aggressiveness on DZA045.5 (Dim3, not shown).

HAC identified three groups among the 50 isolates, according to their average aggressiveness towards the set of *M. truncatula* genotypes (**Figure 3B**). Group 1 clustered all the 19 American isolates from the Us1, Us2 and Us3 pathotypes, as well as the standard Ae109 isolate, which were more aggressive on the *M. truncatula* set of genotypes than the other isolates. Group 2 clustered five American isolates with low to moderate aggressiveness, including two isolates from the Us4 pathotype (LS6 and Ath7), one isolate from the Fr pathotype (MV1), and two isolates with undefined pathotype (MV5 and Ath3). Group 3 consisted of 25 French isolates, one American isolate (MV7) belonging to the Fr pathotype, and the RB84 standard isolate. Isolates from this group showed lower aggressiveness on the *M. truncatula* genotypes. Overall, the mean disease severity observed with all the French isolates was significantly lower than that observed with all the US isolates on the set of *M. truncatula* genotypes (**Figure 1**).

## Discussion

This study investigated the aggressiveness and virulence diversity among pea-infecting *A. euteiches* isolates collected from French and American nurseries. To our knowledge, this study represents the first comprehensive investigation comparing the aggressiveness of *A. euteiches* populations obtained from breeding nurseries across different countries. Results are highly valuable for breeding, particularly because both countries have selected shared sources of resistance (Kraft, 1992; Gritton, 1995) to enhance partial resistance levels in pea varieties.

### Distribution of pathotypes I and III over French and American breeding nurseries

The pea differential set of genotypes employed in this study demonstrated its effectiveness in classifying the sampled isolates into the two main pathotypes, I and III, previously described by Wicker and Rouxel (2001). Other pathotypes were also previously described by these authors, including the avirulent pathotype II on PI1806903 and the avirulent pathotypes IV to XI on at least two of the six pea genotypes, corresponding to generally less aggressive isolates. However, these pathotypes, which often exhibit lower variations between genotypes, were difficult to demonstrate in this study. Our results offer a comprehensive analysis of the prevalence of pathotypes I and III across French and American nurseries. While pathotype III was exclusively found in some American nurseries, pathotype I was detected in both French and American sites. Interestingly, some American nurseries were infested by only one pathotype (pathotype I at Athena and Pullman, pathotype III at Le Sueur), whereas both pathotypes coexisted in the Mount Vernon nursery. At the continent scale, our data show pathogenic variation between American sites, and a much more uniform population structure in France, as described by Wicker and Rouxel (2001). Especially, it is remarkable that pathotype I was found in the three American nurseries located closer to each other (Athena, Mount Vernon and Pullman), but not in the more distant site (Le Sueur).

These results provide valuable insights into the interpretation of previous QTL studies on resistance to *A. euteiches* (Pilet-Nayel et al., 2002; Hamon et al., 2013; Desgroux et al., 2016; Leprévost et al., 2023). These studies identified QTL from pea Recombinant Inbred Line (RIL) and Advanced Backcross (AB) populations, as well as from a pea-Aphanomyces collection, evaluated in six of the seven nurseries sampled in this study (Riec-sur-Belon, Dijon, and Templeux-le-Guérard, FR; Pullman, Athena, and Le Sueur, US). Results of these studies indicated that the resistance QTL showed little specificity across nurseries, which is in line with the predominance of pathotype I in most of them. Indeed, most of the 10 consistent genetic regions identified for resistance to *A. euteiches* in Leprévost et al. (2023) were detected from disease scorings in both the French and US nurseries studied. Particularly, the main *Ae-Ps7.6* QTL was highly consistently detected from disease scores in all FR-US nurseries, except at Mount-Vernon which was not used in QTL mapping studies, on the populations DSP x 90-2131, Baccara x PI180693 and Baccara x 552 RIL. Nevertheless, from disease scoring data in the Le Sueur nursery in which we identified only pathotype III isolates in the present study, another major QTL named *Ae-Ps4.5 (*or *Aph1)*, was detected from the Puget x 90-2079 mapping population (Pilet-Nayel et al., 2002). The source of resistance 90-2079 was derived from the MN313 genotype. MN313 was selected by Davis et al. (1995), from a breeding program that employed screening of progenies in a disease nursery infested with *A. euteiches* in Minnesota, US. The specificity of the major-effect *Ae-Ps4.5* QTL for pathotype III was recently confirmed (Lavaud et al., 2024). The identification of *A. euteiches* isolates within breeding nurseries is essential for understanding the effectiveness of detected QTL and the suitability of selected breeding lines regarding the diversity of pathogen populations. This information is crucial for making informed decisions about their deployment in various pea-growing regions. In France, the presence of a single pathotype simplifies resistance breeding efforts but requires vigilance and precautionary measures against any change in the pathogen population. In contrast, American pea breeders have to consider the presence of both pathotypes of *A. euteiches*, even though pathotype I is predominant, when evaluating their breeding lines. Similarly, in Canada, it is advisable to consider both pathotypes in breeding programs, given that Sivachandra et al. (2021) highlighted the co-occurrence of the two pathotypes, with pathotype I being the predominant one.

Several hypotheses could explain the observed distribution of *A. euteiches* pathotypes in the French and American nurseries studied. Firstly, variations in climatic conditions and sowing dates between the nurseries may account for a part of the pathogen diversity observed. Variation in climate (rainfall, temperature) and growing seasons between the nurseries located in the Pacific Northwest (Pullman, Athena, and Mount-Vernon) and in Minnesota (Le Sueur) regions of the United States may have impacted the dispersion and multiplication of different *A. euteiches* pathogen populations, thus affecting population diversity. In France, fewer climatic and cultural variations were recorded between the three breeding nurseries studied. Secondly, the cultivation of other leguminous crops susceptible to *A. euteiches* in the regions of the nurseries studied might also explain the diversity of *A. euteiches* isolates observed. The large pathogenicity diversity of *A. euteiches* on several legumes including pea, alfalfa, vetch, faba bean, bean and lentil (Malvick and Percich, 1998; Levenfors et al., 2003; Moussart et al., 2008; van Leur et al., 2008) and the genetic variation found even within fields (Grünwald and Hoheisel, 2006) suggest that this pathogen has the ability to adapt to different cropping systems and rotations. Our pathogenicity characterization results for French and American isolates on *M. truncatula* genotypes provide further support for this last hypothesis.

### Aggressiveness of French and American isolates on *M. truncatula*

The set of differential genotypes of *M. truncatula* especially curated for this study made it possible to highlight the level of adaptation of isolates from French and American nurseries to a host model legume genetically close to cultivated alfalfa. Our results revealed that isolates from French nurseries were less aggressive on *M. truncatula* but more aggressive on pea compared to most isolates from American nurseries. The set of *M. truncatula* genotypes grouped isolates by geographical origin, with lower variability in aggressiveness observed among the French isolates compared to the American isolates. These results suggest that *A. euteiches* isolates from American nurseries display greater adaptation to *Medicago* spp., while isolates from French nurseries are stronger adapted to pea. The greatest adaptation of American isolates to *Medicago* spp. could be attributed to the extensive and longstanding cultivation of this crop in the north-central regions of the United States, which is the world’s leading producer of *Medicago* spp. In contrast, *Medicago* spp. cultivation is relatively recent and limited to specific regions in France. Even if *Medicago* spp. are native to Europe, no species of the genus *Medicago* were cultivated in any of the three French nurseries studied, whereas alfalfa has been grown in rotation with pea in the United States over the past few decades, particularly in areas such as the Midwest region, including states like Minnesota. Historical literature reveals that alfalfa was the earliest forage crop cultivated in the USA, while France focused on its production in the 1950s, mainly for dehydrated alfalfa (Goplen et al., 1987). Thus, the localized selection of *A. euteiches* populations by pea in France may have resulted in an increased specialization of isolates on this host. In contrast, the more diverse legume rotation practices in America may have facilitated the presence of isolates with markedly different host pathogenicity. The reduced pathogenicity diversity observed in French isolates in comparison to American ones can likely be attributed to a more uniform selection pressure resulting from the predominant cultivation of pea as the main leguminous crop in France. Since the 1980s, the prevalence of pea crops and the susceptibility of the pea cultivars used by growers in France may account for the high aggressiveness and limited variation in pathogenicity observed among the French isolates, as suggested by Quillévéré-Hamard et al. (2018).

Our results suggest that the plant host plays a key role in driving the evolution of *A. euteiches* populations, offering promising perspectives for exploiting the host as a means to manage pathogen populations. The cultivation of diversified legume hosts in rotation with peas or alfalfa could potentially help limit the adaptation or even the size of *A. euteiches* populations. In France, the growing of faba bean, resistant to *A. euteiches* (Moussart et al., 2008), in alternation with pea, has been recommended for several years as an effective strategy to improve the management of Aphanomyces root rot.

## Data availability statement

The original contributions presented in the study are included in the article/**Supplementary Material**. Further inquiries can be directed to the corresponding authors.

## Author contributions

AM: Conceptualization, Data curation, Formal analysis, Investigation, Methodology, Writing – original draft. CLa: Data curation, Formal analysis, Methodology, Validation, Visualization, Writing – review & editing. CO: Data curation, Formal analysis, Methodology, Writing – review & editing. TL: Formal analysis, Methodology, Software, Writing – review & editing. M-LP-N: Conceptualization, Formal analysis, Investigation, Supervision, Validation, Writing – review & editing. CLM: Conceptualization, Formal analysis, Investigation, Supervision, Validation, Writing – review & editing.

## Supplementary material

Additional File 1 Mean weather conditions (mean temperature and rainfall) recorded in the different French (Templeux-Le-Guérard, Dijon, and Riec-sur-Belon) and American (Athena, OR; Mount Vernon, WA; Le Sueur, MN; and Pullman, WA) nurseries during the last twenty years (http://www.infoclimat.fr.html and http://www.prism.oregonstate.edu for French and American nurseries, respectively).

Additional File 2 Bar plot of disease severity (EMMeans) **(A)** on the six differential pea genotypes, for the 51 *A. euteiches* isolates and two *A. euteiches* reference isolates (RB84 and Ae109) classified into pathotypes I and III according to Wicker and Rouxel (2001), and **(B)** on the five differential *M. truncatula* genotypes, for 50 *A. euteiches* isolates and two *A. euteiches* reference isolates (RB84 and Ae109) classified into pathotypes Fr, Us1, Us2, Us3 and Us4 in this study. NA: isolates with undefined pathotype.
