# Supplementary material for: Pathotype characterization of Aphanomyces euteiches isolates collected from pea breeding nurseries
Source: Front Plant Sci. 2024 Mar 28;15:1332976. doi: 10.3389/fpls.2024.1332976 (PMC11007135; doi:10.3389/fpls.2024.1332976)
Supplement: Additional File 1 — Mean weather conditions (mean temperature and rainfall) recorded in the different French (Templeux-Le-Guérard, Dijon, and Riec-sur-Belon) and American (Athena, OR; Mount Vernon, WA; Le Sueur, MN; and Pullman, WA) nurseries during the last twenty years (http://www.infoclimat.fr.html and http://www.prism.oregonstate.edu for French and American nurseries, respectively). [file Table_1.doc]

**Additional File 1**

|  |  | **French nurseries** | | | **American nurseries** | | | |
| --- | --- | --- | --- | --- | --- | --- | --- | --- |
|  |  | **Dijon** | **Riec-sur-Belon** | **Templeux-Le Guérard** | **Athena** | **Mount-Vernon** | **Le Sueur** | **Pullman** |
| **Crop information** | Sowing date | Mid-February | Mid-February | Mid-February | Mid-March | Mid-March | Mid-April to May | Mid-April |
|  | Cropping season | February-July | February-July | February-July | Mid-March to mid-July | Mid-March to mid-July | Mid-April to mid-July | Mid-April to August |
| **Mean Temperature (°C)** | March | 6.8 | 7.6 | 6.6 | **16.3** | **7.5** | **-0.8** | **-0.8** |
|  | April | 9.4 | 7.6 | 9.0 | **19.3** | **13.7** | **7.2** | **7.2** |
|  | May | 13.8 | 10.1 | 13.1 | **23.3** | **18.1** | **14.7** | **20.5** |
|  | June | 16.9 | 15.8 | 15.3 | **26.2** | **23.2** | **19.9** | **23.4** |
|  | July | 19.7 | 21.8 | 17.9 | **27.6** | **25.6** | **21.9** | **25.1** |
| **Rainfall (mm)** | March | 43.7 | 40.7 | 44.7 | 119.4 | 124.5 | 119.4 | 40.6 |
|  | April | 48.9 | 60.5 | 44.8 | 58.4 | 73.7 | 58.4 | 63.5 |
|  | May | 74.2 | 56.9 | 47.4 | 63.5 | 88.9 | 63.5 | 86.4 |
|  | June | 56.1 | 46.4 | 59.4 | 167.6 | 94.0 | 167.6 | 129.5 |
|  | July | 59.6 | 40.5 | 45.2 | 185.4 | 96.5 | 185.4 | 106.7 |
