# Supplementary figures and images for: Pathotype characterization of Aphanomyces euteiches isolates collected from pea breeding nurseries
Source: Front Plant Sci. 2024 Mar 28;15:1332976. doi: 10.3389/fpls.2024.1332976 (PMC11007135; doi:10.3389/fpls.2024.1332976)

A

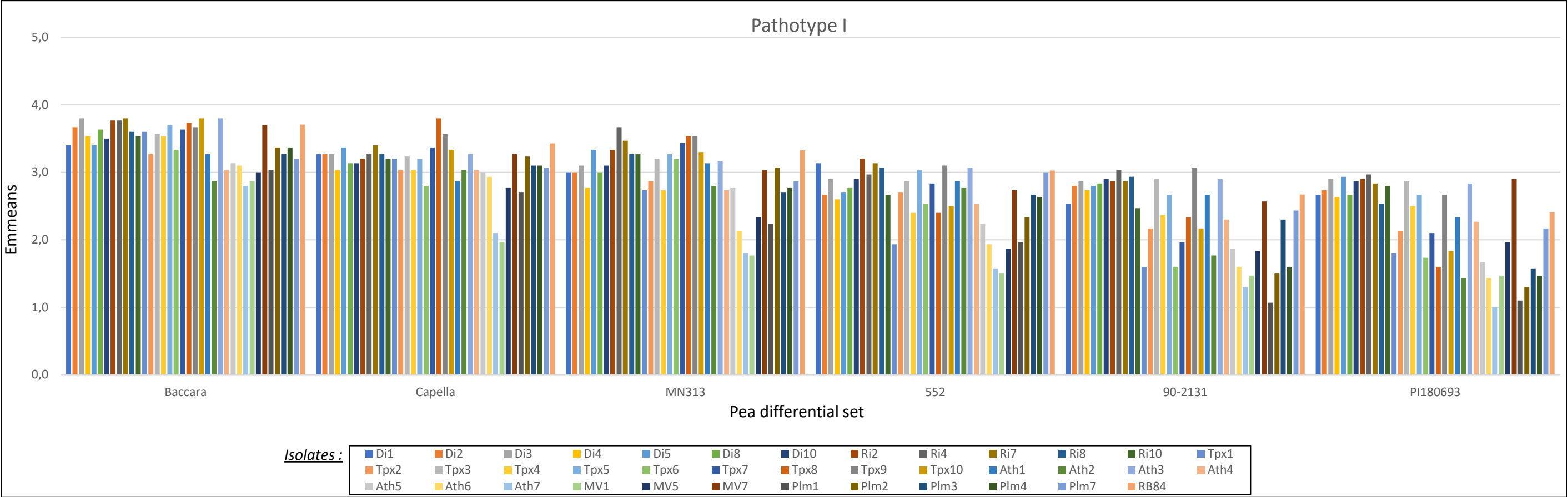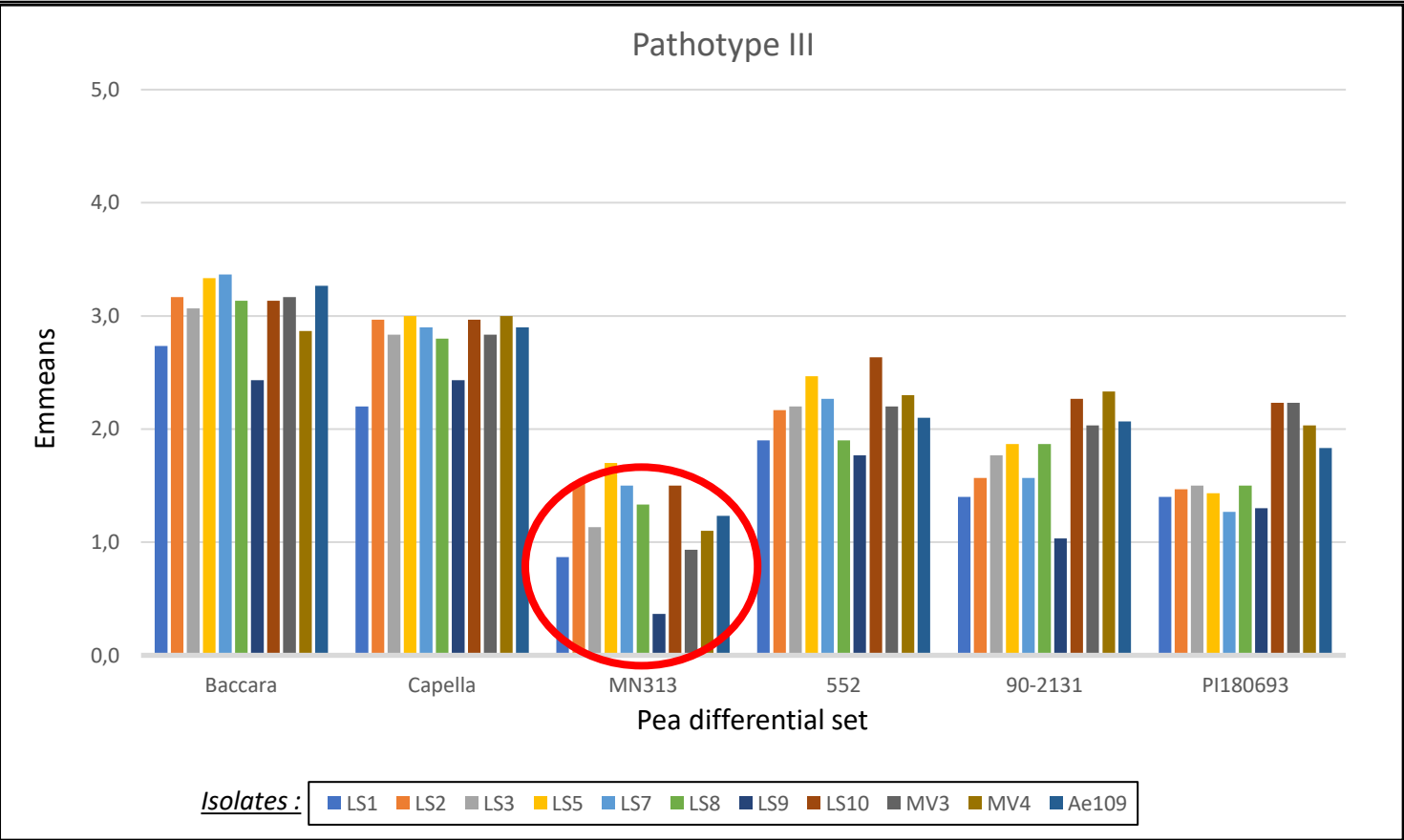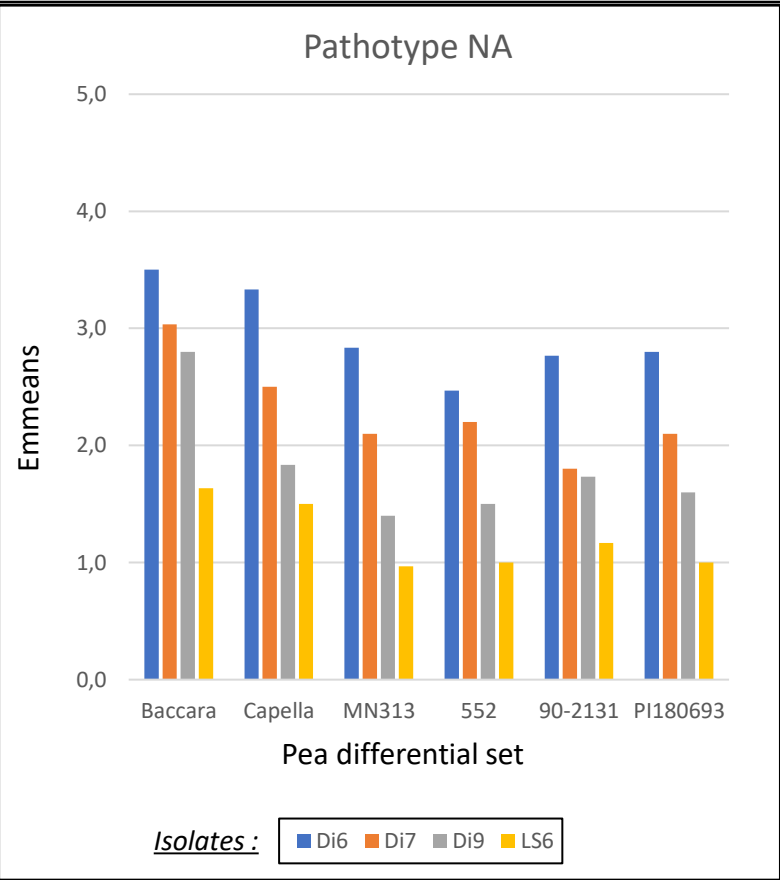

B

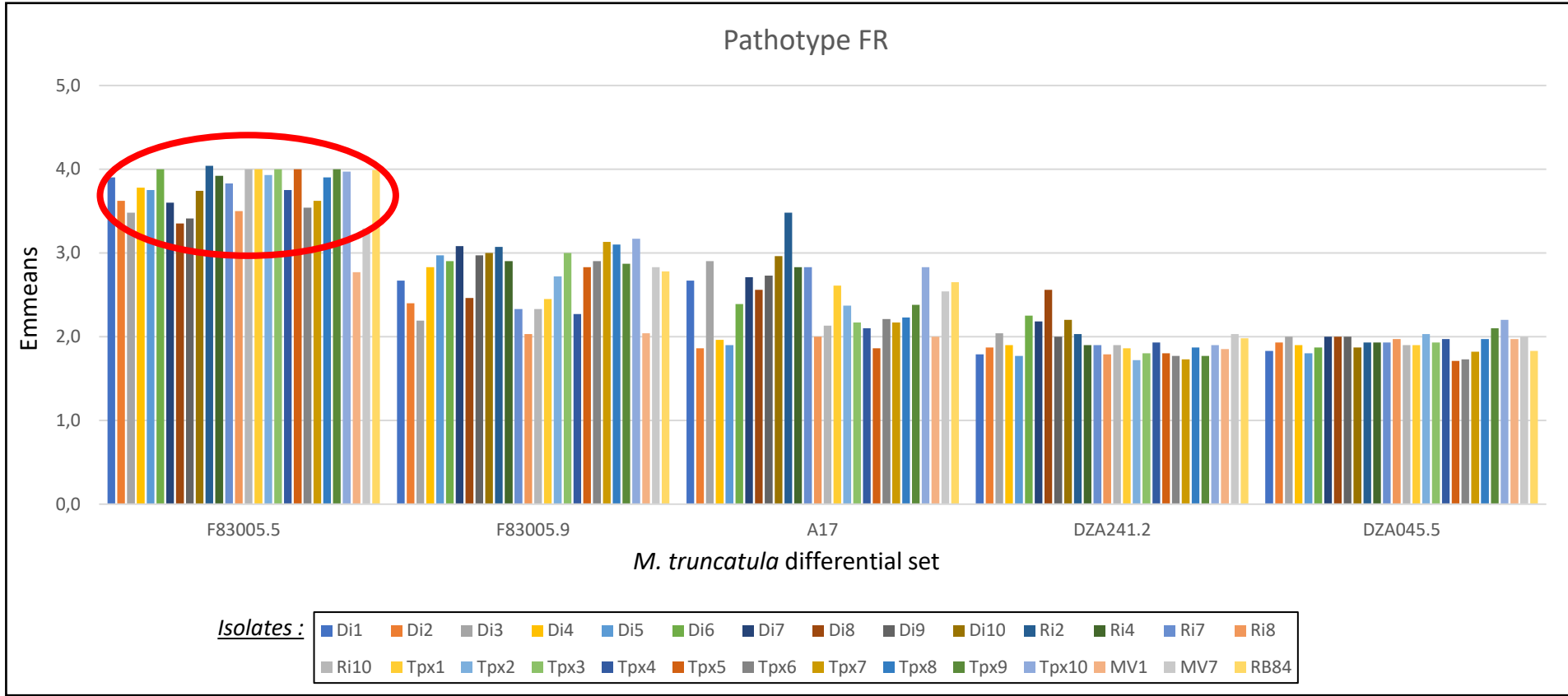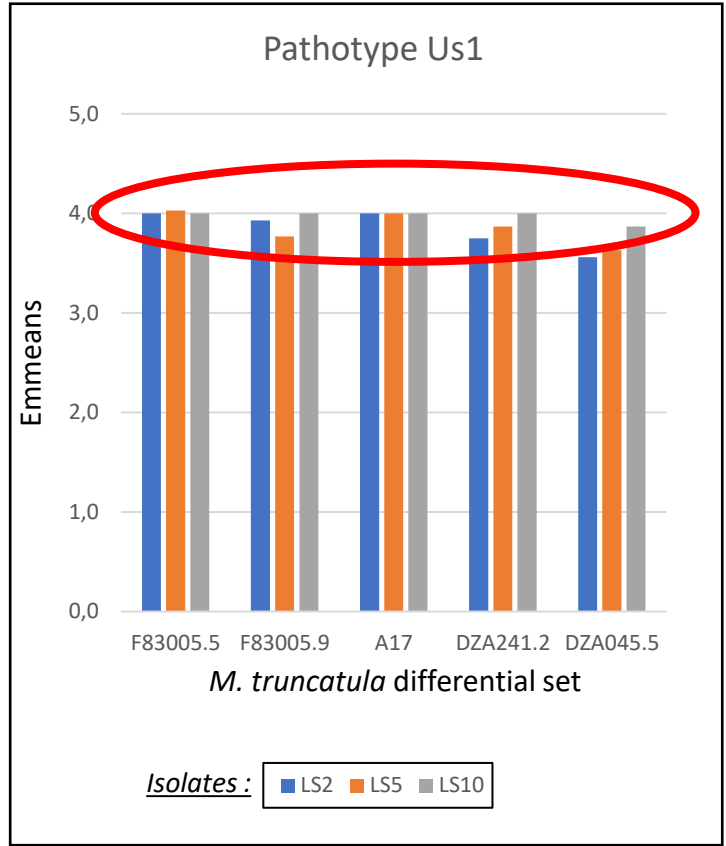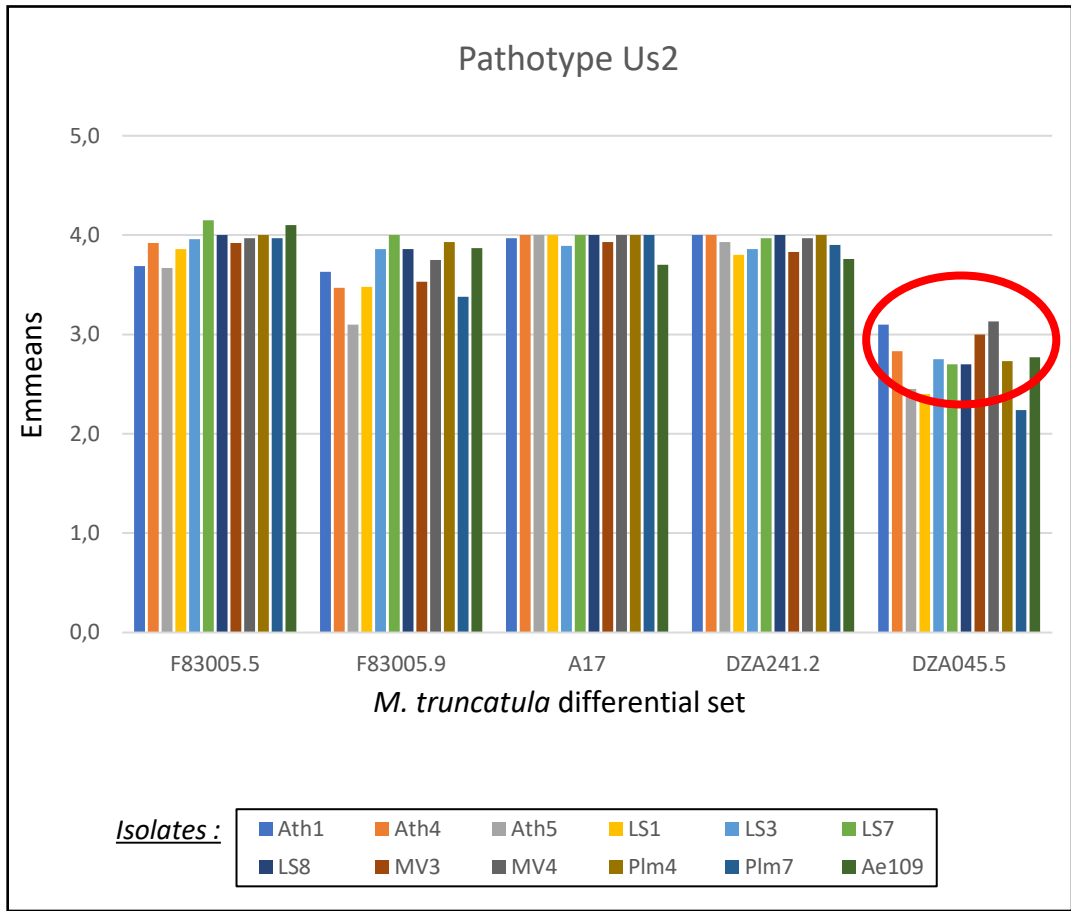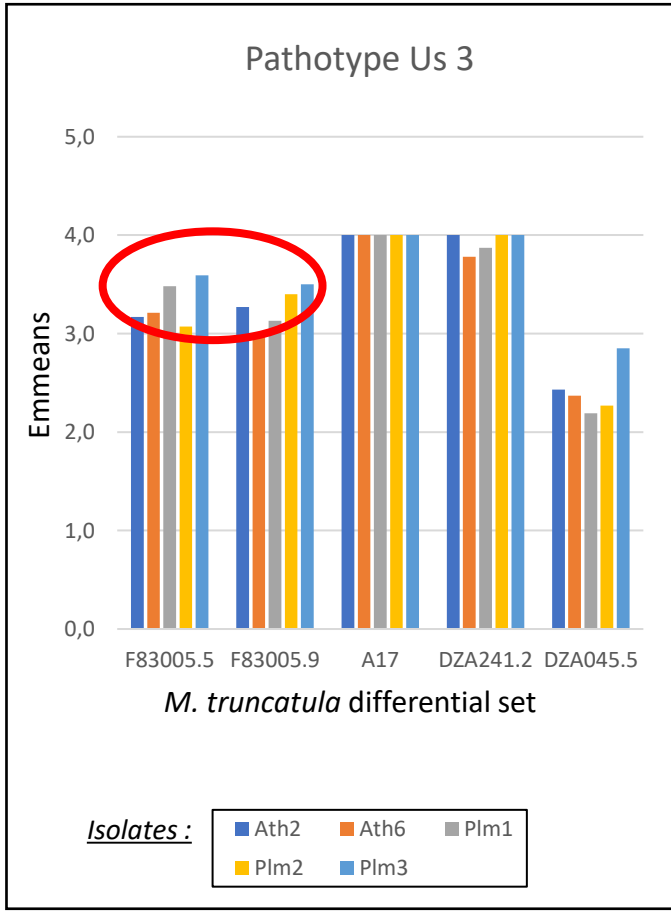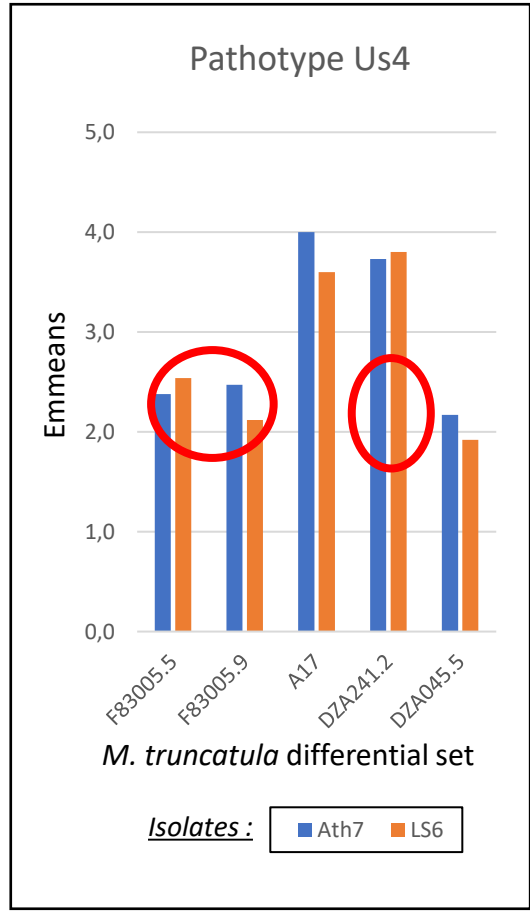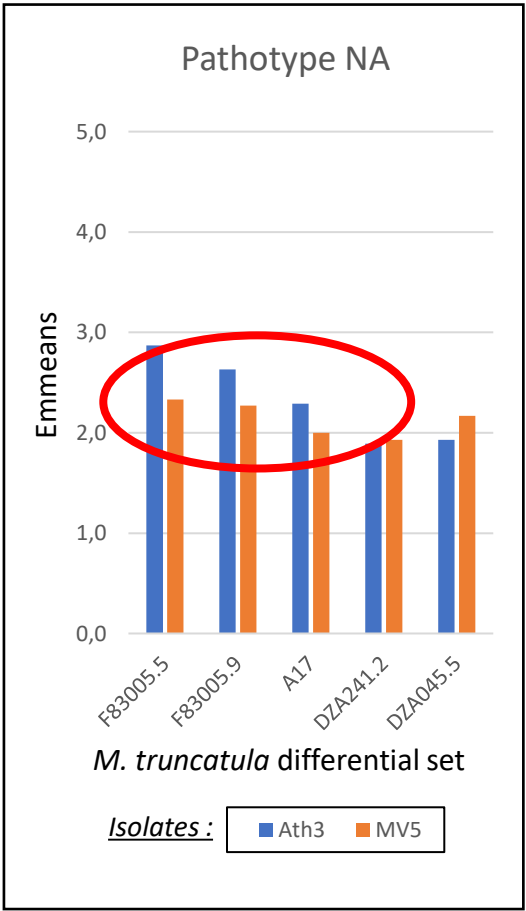

Supplement: Additional File 2 — Bar plot of disease severity (EMMeans) (A) on the six differential pea genotypes, for the 51 A. euteiches isolates and two A. euteiches reference isolates (RB84 and Ae109) classified into pathotypes I and III according to Wicker and Rouxel (2001), and (B) on the five differential M. truncatula genotypes, for 50 A. euteiches isolates and two A. euteiches reference isolates (RB84 and Ae109) classified into pathotypes Fr, Us1, Us2, Us3 and Us4 in this study. NA: isolates with undefined pathotype. [file DataSheet_1.pdf]
